# Doubly-Charged Negative Ions as Novel Tunable Catalysts: Graphene and Fullerene Molecules Versus Atomic Metals

**DOI:** 10.3390/ijms21186714

**Published:** 2020-09-13

**Authors:** Kelvin Suggs, Alfred Z. Msezane

**Affiliations:** Department of Physics and CTSPS, Clark Atlanta University, Atlanta, GA 30314, USA; spinmatrix1@gmail.com

**Keywords:** Graphene, fullerenes, atomic metals, doubly-charged anions, tunable catalysts, electron scattering, water oxidation

## Abstract

The fundamental mechanism underlying negative-ion catalysis involves bond-strength breaking in the transition state (TS). Doubly-charged atomic/molecular anions are proposed as novel dynamic tunable catalysts, as demonstrated in water oxidation into peroxide. Density Functional Theory TS calculations have found a tunable energy activation barrier reduction ranging from 0.030 eV to 2.070 eV, with Si^2−^, Pu^2−^, Pa^2−^ and Sn^2−^ being the best catalysts; the radioactive elements usher in new application opportunities. C_60_^2−^ significantly reduces the standard C_60_^−^ TS energy barrier, while graphene increases it, behaving like cationic systems. According to their reaction barrier reduction efficiency, variation across charge states and systems, rank-ordered catalysts reveal their tunable and wide applications, ranging from water purification to biocompatible antiviral and antibacterial sanitation systems.

## 1. Introduction

The importance of the need to understand the fundamental functional roles of the various constituents of current complicated catalysts is demonstrated in a recent investigation of water oxidation catalyzed by the oxygen-evolving complex of photosystem II [1]. These authors also pursue the development of new artificial water oxidation catalysts for artificial photosynthetic water oxidation [2]. In recent years, research on water oxidation catalyzed by various systems, processes and mechanisms has intensified [3,4,5,6,7,8,9,10,11,12,13,14,15,16]. The advent of the COVID-19 pandemic has also accelerated a vigorous search for and synthesis of atomic nanoparticles and catalysts for various applications (see for examples [17,18]). The stability of organic solar cells that is required before their actual commercialization is of particular interest here [19]. The use of fullerenes with large electron affinities (EAs) is part of the recommended solution [19]. The formation of high-energy singlet oxygen and super oxygen radical anions, contributing to the degradation mechanism of the employed polymers, could be mitigated through the use of appropriate tunable catalysts, presented in this paper.

Here, we propose using doubly-charged atomic/molecular negative ions as novel tunable catalysts for general applications. Their effectiveness is demonstrated in the catalysis of water oxidation into peroxide. The fundamental mechanism underlying negative-ion catalysis involves anionic molecular complex formation in the transition state (TS), with the atomic/molecular negative ion breaking up the hydrogen bond strength. The oxidation reaction to be catalyzed that is considered here is 2 H_2_O + O_2_ → 2 H_2_O_2_, as opposed to that discussed in [1], 2 H_2_O → H_2_O_2_ + H_2_. Here, we first introduce the anionic catalyst, Au^−^, to break up the hydrogen-bond strength in the water and then follow with the addition of O_2_ to form the desired H_2_O_2_. This process can be contrasted with the well-investigated muon (μ)-catalyzed nuclear fusion cycle, which involves the resonant formation of the muonic molecular ion, *dt*μ [20], with *d* and *t* being the deuteron and the triton, respectively. Consequently, this paper investigates the energy barrier reduction during water oxidation catalyzed by various doubly-charged negative atomic/molecular ions, with Z varying from 14 through the lanthanide atoms to the interesting radioactive actinides. The purely radioactive element 85, At, is also included; its EA was measured in a recent breakthrough experiment [21]. 

The Royal Society of Chemistry published the themed collection “Single Atoms as Catalysts” [22] to celebrate the International Year of the Periodic Table. Naturally, this has been followed by the investigation of single fullerene negative ions as catalysts [23]. Their effectiveness has been demonstrated in the catalysis of water oxidation into peroxide and in water synthesis from H_2_ and O_2_ using the fullerene anions from C_44_^−^ to C_136_^−^. Density Functional Theory (DFT) calculations found the C_60_^−^ anion to be optimal; namely, it reduces the TS energy barrier by the same amount in both the oxidation of water into peroxide and in the water synthesis from H_2_ and O_2_. Importantly, DFT also found the C_136_^−^ anion to represent the best catalyst for both water oxidation into peroxide and water synthesis from H_2_ and O_2_. 

Recently, we explored the oxidation of water into peroxide using the doubly-charged standard reference C_60_^2−^ anion, the negative metal ions Sn^2−^, Pd^2−^, Ag^2−^ and Au^2−^, and some lanthanide and actinide anions [24]. The selection of the Sn^2−^, Pd^2−^ and Au^2−^ anions was motivated by the successful experiments [25,26,27] relating to the catalysis of H_2_O_2_ formation from H_2_ and O_2_ using Au, Pd and Sn nanoparticles. Preliminary DFT TS calculations found Pu^2−^, Pa^2−^, Sn^2−^ and La^2−^ to be the best catalysts among the investigated anions in reducing considerably the energy barrier. In [1], the role of the Ca^2+^ was elucidated as well. Previously, cationic systems were discovered to increase the TS energy barriers in the synthesis of peroxide from H_2_O [28]. This could render them essential as inhibitors in controlling and regulating catalysis. 

Two experiments provide an excellent understanding of the fundamental mechanism involved in negative ion catalysis [29,30]. Also, the interplay between Regge resonances and Ramsauer–Townsend minima in the electron elastic total cross sections (TCSs) has, along with the large EAs, been identified as the mechanism underlying negative ion catalysis [31]. In [29], the vertical detachment energies (VDEs) of the Au^−^M complexes (M = Ne, Ar, Kr, Xe, O_2_, CH_4_ and H_2_O) were investigated. A stronger interaction between the Au^−^ anion and H_2_O, as well as between the Au^−^ and the CH_4_ molecule, was found. However, a weaker interaction was observed between the Au^−^ anion and O_2_, as well as with the noble gases Ne, Ar, Kr and Xe. Indeed, the anionic complexes Au^−^(H_2_O)_2_ and Au^−^(H_2_O)_1_ have been characterized as anionic Au^−^ interacting with two and one water molecules, respectively [30]. A similar analysis is applicable to the anionic molecular complex Au^−^(CH_4_). 

## 2. Results

### 2.1. Reaction Dynamics

Following [31], in this study we first consider the slow oxidation process of water to peroxide without negative-ion catalysts. We use the Au^−^ anionic catalyst to illustrate the mechanism in the reaction:

2 H_2_O + O_2_ → 2 H_2_O_2_ (1)

Then, we apply the Au^−^ anion to speed up the reaction (1) and obtain:

Au^−^(H_2_O)_2_ + O_2_ → Au^−^ + 2 H_2_O_2_ (2)

Au^−^ + 4 H_2_O + O_2_ → Au^−^(H_2_O)_2_ + 2 H_2_O_2_ (3)

Add reactions (2) and (3) and obtain:

4 H_2_O + 2 O_2_ → 4 H_2_O_2_ (4)

Similar results as in (4) are obtained when the Au^−^ anion is replaced by the various negative-ion catalysts considered here. The question we want to address here is which of the negative-ion catalysts speed up the reaction. Furthermore, is a doubly-charged negative ion catalyst more effective in the catalysis of water into peroxide or vice versa? Importantly, the experiment [29] found a stronger interaction between the Au^−^ and H_2_O and a weaker one between Au^−^ and O_2_. Indeed, the Au^−^(H_2_O)_1,2_ anionic molecular complexes formation in the TS was identified as the mechanism for breaking up the hydrogen bonding strength in water during catalysis. 

### 2.2. Electron Scattering Total Cross Sections and Electron Affinity Calculations

**Figure 1:**
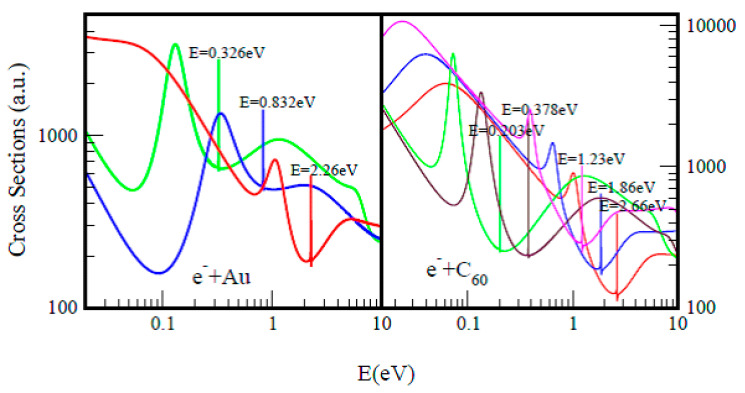
The standard total cross sections (TCSs) (a.u.) for electron elastic scattering from atomic Au (left panel) and the fullerene molecule C_60_ (right panel) are contrasted. For atomic Au, the red, blue and green curves represent TCSs for the ground, metastable and excited states, respectively. For the C_60_ fullerene, the red, blue and pink curves represent TCSs for the ground and the metastable states, respectively, while the brown and green curves denote TCSs for the excited states. The dramatically sharp resonances in both figures correspond to the Au^−^ and C_60_^−^ negative ions formation during the collisions.

**Figure 2:**
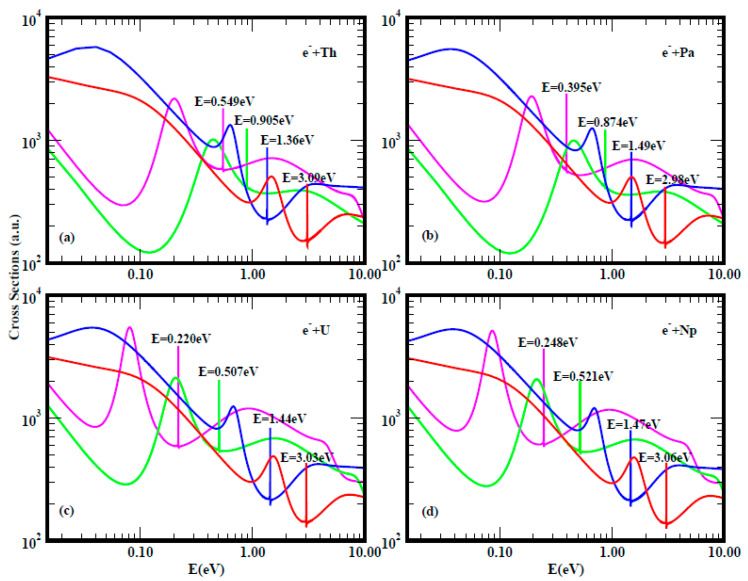
Total cross sections (a.u.) for electron elastic scattering from atomic Th(a), Pa(b), U(c) and Np(d). The red, blue, green and pink curves represent TCSs for the ground, metastable and two excited states, respectively. The dramatically sharp resonances in the figures correspond to the Th^−^, Pa^−^, U^−^ and Np^−^ negative ions formation during the collisions.

**Figure 3:**
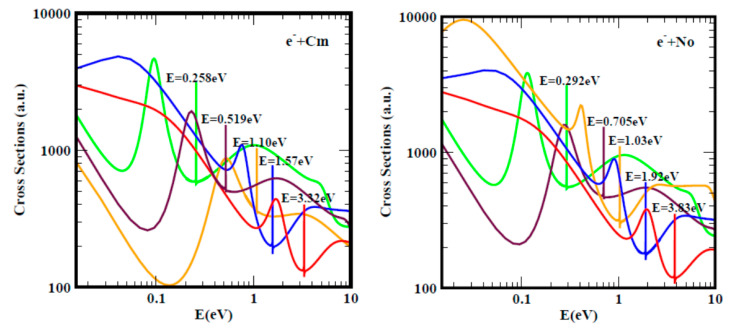
The total cross sections (a.u.) for electron elastic scattering from atomic Cm (left panel) and No (right panel) are contrasted. For both Cm and No, the red, blue and orange curves represent TCSs for the ground and metastable states, respectively. The brown and green curves denote TCSs for the excited states. The dramatically sharp resonances in both figures correspond to the Cm^−^ and No^−^ negative ions formation during the collisions.

For a better understanding and appreciation of the results presented here, namely doubly-charged negative ions as a novel tunable mechanism for catalysis, we first present, in Figure 1, Figure 2 and Figure 3, Regge-pole calculated low-energy electron scattering TCSs, focusing on large atoms such as the actinides. These are difficult to handle experimentally because of their radioactive nature. The interplay between Regge resonances and Ramsauer–Townsend (R–T) minima calculated through our Regge pole methodology has been identified as the fundamental atomic mechanism underlying nanoscale catalysis. Anionic catalysis involves the breaking up of molecular bonds in the transition state (TS) [31]. The R–T minimum provides an excellent environment and mechanism for breaking up molecular bonds in new molecule creations as well as in anionic catalysis [31]. Generally, calculated low-energy electron elastic TCSs are characterized by dramatically sharp resonances that manifest ground, metastable and excited negative ion formation, with the ground state binding energies (BEs) yielding the important EAs that are theoretically challenging to calculate for complex heavy systems.

In Figure 1, the standard elastic TCSs for the Au and C_60_ systems taken from [32] are displayed. They reveal the important characteristic negative ion BEs, R–T minima and shape resonances. It is noted here that the extracted anionic BEs from the ground state TCSs for Au and C_60_ are in outstanding agreement with the measured EAs for Au [30,33,34] and for C_60_ [35,36,37]. Figure 2 presents the Regge-pole calculated TCSs for the radioactive Th, Pa, U and Np actinide atoms. With the exception of the Th atom, no measurements of the EAs are available for any actinide atom. Very recently, the EAs of At was measured [21], as well as that of atomic Th [38]. However, the latter EA value has been identified with the BE of an excited state of Th [39]. Figure 3 compares the Regge-pole calculated TCSs for the large actinide atoms Cm and No. These TCSs are also generally characterized by negative ion formation, shape resonances and R–T minima, as well as exhibiting atomic and fullerene molecular near-threshold behavior [40]. Furthermore, a polarization-induced metastable cross section with a deep R–T minimum near the threshold is identified in the Cm TCSs. In the No TCSs, this minimum has flipped over to a shape resonance that appears very close to the threshold. We attribute these novel manifestations to size effects and to the orbital collapse significantly impacting the polarization interaction.

Importantly, the characteristic sharp peaks in the TCSs of these systems could be used to catalyze various reactions, including enzymes with VDEs within the BEs and the R–T minima of the negative ion catalyst. Additionally, these results should help experiments and theories probe the structure and dynamics of these poorly studied and interesting actinide atoms.

### 2.3. Transition State Energy Barriers Calculations

**Figure 4:**
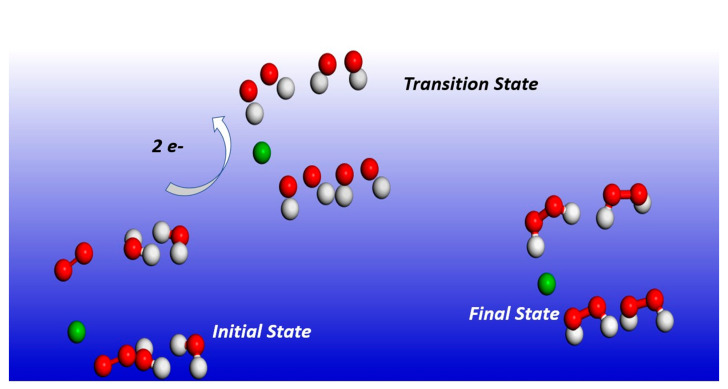
Doubly-charged anionic transition state geometry optimization for Sn. The red, white and green spheres represent O_2_, H_2_ and Sn (catalyst), respectively. Note the broken bonds in the transition state.

**Figure 5:**
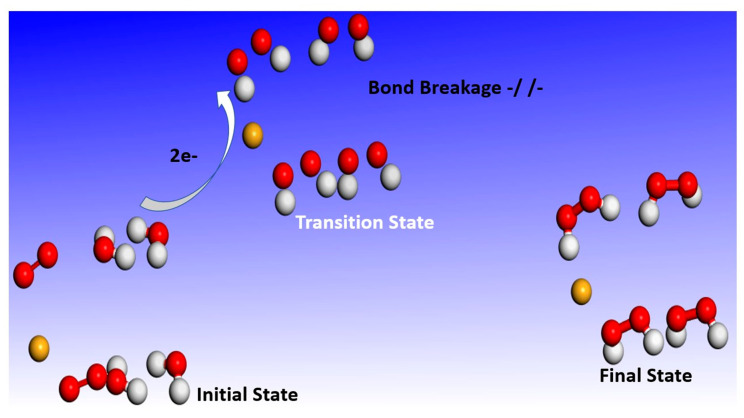
Doubly-charged anionic transition state geometry optimization for Pu. The red, white, and gold spheres represent O_2_, H_2_ and Pu (catalyst), respectively. Note the broken bonds in the transition state.

**Figure 6:**
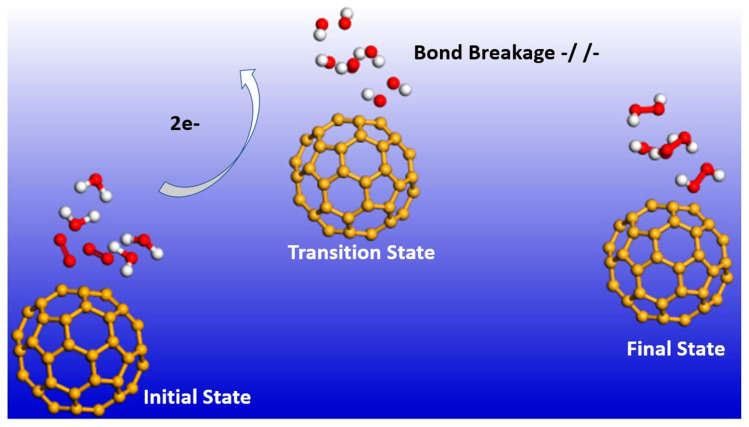
Doubly-charged anionic transition state optimization for C_60_-6. The red, white and gold spheres represent O_2_, H_2_ and C_60_ (catalyst), respectively. Note that, here as well, the bonds are broken in the transition state.

**Figure 7:**
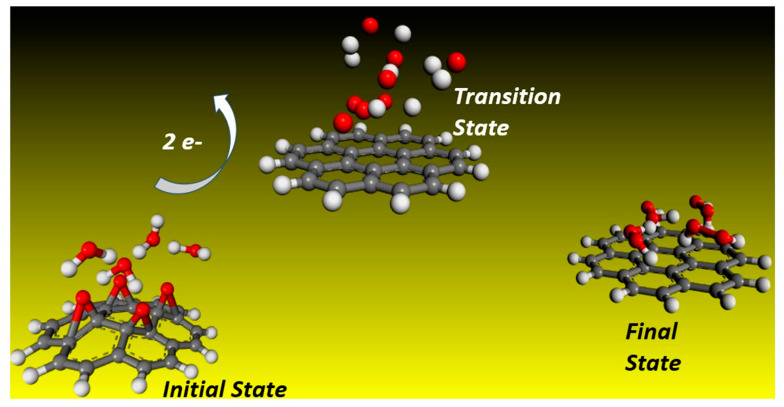
Geometrically optimized molecules of doubly-charged graphene Gr24-6 catalyzing the water conversion to peroxide, indicated by initial, transition and final states. Carbon, oxygen and hydrogen are represented by the gray, red and white spheres, respectively. Note, here as well, that the bonds are broken in the transition state.

It has already been pointed out in the introduction that the fundamental mechanism underlying negative-ion catalysis involves anionic molecular complex formation in the TS, with the atomic/molecular negative ion breaking up the bond strength. Doubly-charged anionic transition state geometry-optimized calculations of the Sn and Pu catalysts are shown in Figure 4 and Figure 5, respectively. Figure 6 and Figure 7 provide the results for C60-6 and for graphene, Gr24-6, respectively. DFT and dispersion-corrected DFT approaches have been employed for the transition state evaluations. The important communication that is conveyed here by the figures is the breakage of bonds in the transition state by the doubly-charged catalysts.

**Figure 8:**
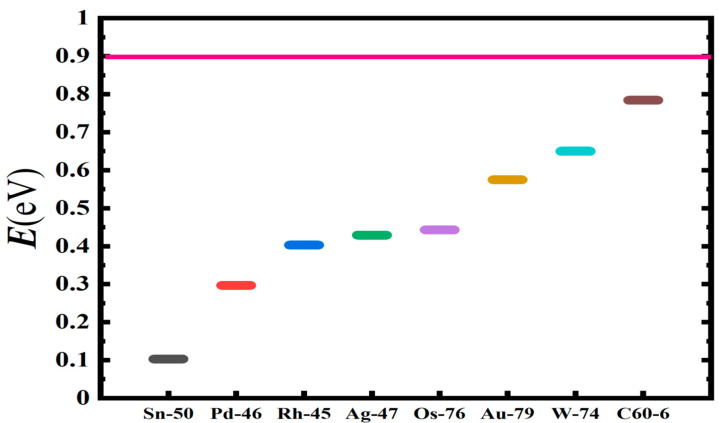
Transition state energy barrier reduction, E(eV), for selected doubly-charged anionic systems of popular catalysts in ascending order of catalytic effectiveness. The horizontal axis shows the not-to-scale catalyzing systems. The pink line represents the energy position with no catalyst present.

**Figure 9:**
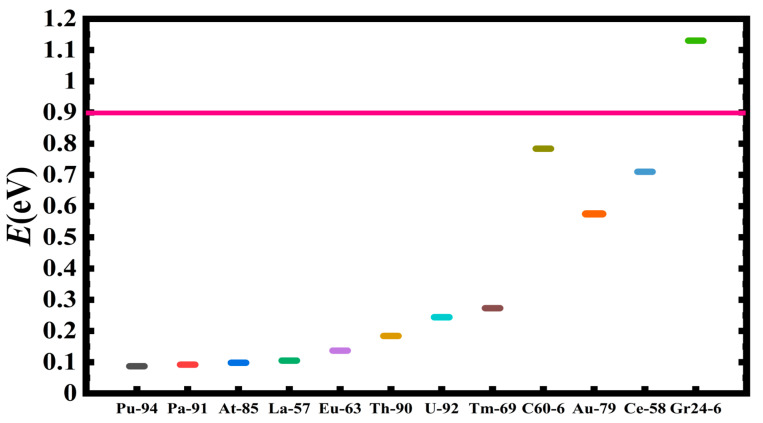
Transition state energy barrier reduction, E(eV), in ascending order of catalytic effectiveness for doubly-charged heavy systems, including C_60_ and 24 carbon graphene (Gr24-6). The horizontal axis indicates the not-to-scale catalyzing systems, and the solid pink line represents the barrier energy position with no catalyst present.

**Figure 10:**
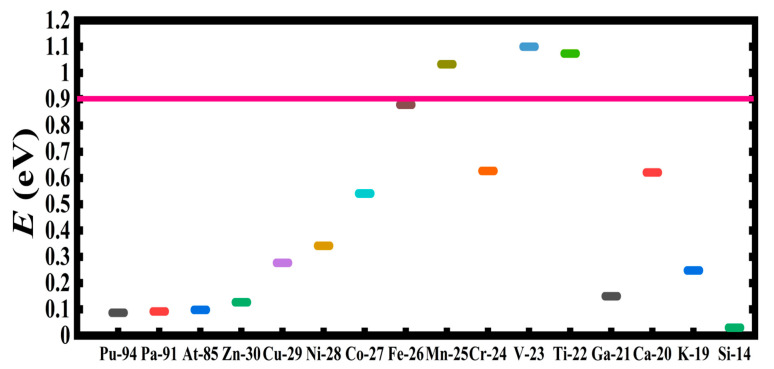
Transition state energy barrier reduction, E(eV), in ascending and descending order of catalytic effectiveness for doubly-charged atomic systems. The relative effectiveness of the popular components of catalysts from Z-30 to K-19 is emphasized. For the high Zs, the radioactive atoms are the most effective, while for the small ones, Si is the best doubly-charged catalyst. The horizontal axis reflects the catalyzing systems, and the solid pink line shows the barrier energy position with no catalyst present.

Table 1 presents a rank-ordered comparison of the activation energy barrier reduction by the doubly-charged systems, DCS(-2). These vary from the smallest investigated atom Si^2−^ with an energy value of 0.030 eV to the largest atom U^2−^ with an energy value of 0.244 eV. For comparison, Table 1 also includes the doubly-charged fullerene C_20_, C_60_ and C_136_, as well as the graphene Gr24-6 molecules. Indeed, the lowest energy reduction results from Si^2−^. It is followed by the three radioactive elements Pu^2−^, Pa^2−^ and At^2−^, with Sn^2−^ and La^2−^ close by with a value of about 0.1 eV. Clearly, the radioactive anions promise the realization of a dynamic tunable catalyst for the oxidation of water into peroxide without the need of an external power input. A visualization of the results is conveyed by Figure 8, Figure 9 and Figure 10. These figures can also be used to select the appropriate doubly-charged systems for the oxidation of water into peroxide, as well as to model more complex catalytic systems.

Table 2 contrasts the activation energy barrier reduction by the doubly-charged systems, DCS(-2), with those by the singly-charged systems, SCS(-1). Generally, the DCS(-2) and SCS(-1) energies are close to each other for many of the systems that are considered here, implying that the SCS(-1) systems are already charge-optimized. However, for Au and C_136_, the DCS(-2) energies are greater than those of the SCS(-1) by factors of about 1.44 and 2.11, respectively. This implies that decreasing the charge by –1 on the Au and C_136_ systems reduces their catalytic effectiveness, meaning that the energy barrier is increased by this change of charge. For Pa, Sn, Ca and C_60_, the SCS(-1) values are greater than those of the DCS(-2) by factors of about 3.54, 11.75, 1.91 and 2.50, respectively. Indeed, the decrease of the charge by –1 in these systems increases their catalytic effectiveness by factors varying from 1.91 for Ca to 11.75 for Sn, allowing them to be charge-tunable.

The results in Table 2 demonstrate that, in an environment where the charge on the systems could change, their catalytic effectiveness could increase or decrease. It is further noted that the catalytic effectiveness of C_20_ and graphene exhibit a charge stability; namely, they remain unchanged when the charge changes from –1 to –2 or vice versa. It can now be understood why the graphene molecule sometimes needs assistance from other atoms for an effective catalysis.

## 3. Method of Calculation

### 3.1. Total Cross Sections Calculation

Regge poles, singularities of the S-matrix, rigorously define resonances [41,42], and in the physical sheets of the complex plane they correspond to bound states [43]. Indeed, it has been confirmed that Regge poles formed during low-energy electron elastic scattering become stable bound states [44]. Being generalized bound states, they can be used to reliably calculate the BEs of the ground, metastable and excited states of the formed negative ions during the collision of an electron with complex heavy systems through the TCSs calculations. Here, we adopt the Regge-pole methodology, also known as the complex angular momentum (CAM) method, for the calculation of the electron scattering TCSs. The near-threshold electron–atom/fullerene collision TCS resulting in negative ion formation is calculated using the Mulholland formula [45]. In the form below, the TCS fully embeds the essential electron-electron correlation effects [46,47] (atomic units are used throughout):

(5) σtot(E)  =  4πk−2∫0∞Re[1−S(λ)]λdλ−8π2k−2∑nImλnρn1 + exp(−2πiλn) + I(E)

In Equation (5), S(λ) and λ are, respectively, the S-matrix and the CAM, $k=\sqrt{2mE}$, *m* being the mass and *E* the impact energy, *ρ_n_* is the residue of the S-matrix at the *n*^th^ pole, λ*_n_*, and *I*(*E*) contains the contributions from the integrals along the imaginary λ-axis; its contribution has been demonstrated to be negligible [48].

As in [49], the complicated details of the electronic structure of the atom/fullerene itself are not considered here. The incident electron is assumed to interact with the complex atom/fullerene through the Thomas–Fermi type potential, known as the Avdonina, Belov and Felfli (ABF) potential [50], which accounts for the vital core-polarization interaction.

(6) $$U(r)=-\frac{Z}{r(1+\alpha Z^{1/3}r)(1+\beta Z^{2/3}r^{2})}$$

In Equation (6), *Z* is the nuclear charge, and *α* and *β* are the variation parameters. This potential has the appropriate asymptotic behavior, *viz.* ~ −1/(αβr^4^), and accounts properly for the crucial polarization interaction at low energies. Extensively studied [51], the potential in Equation (6) has five turning points and four poles connected by four cuts in the complex plane. The presence of the powers of Z as coefficients of *r* and *r*^2^ in Equation (6) ensures that spherical and nonspherical atoms/fullerenes, as well as large and small systems, are correctly treated. The effective potential

(7) $$V(r)=U(r)+\lambda (\lambda +1)/2r^{2}$$

is considered here as a continuous function of the variables *r* and complex *λ*. The details of the calculations may be found in [52].

In the calculations, the optimal value of *α* was determined to be 0.2. When the TCS as a function of *β* has a dramatically sharp resonance [48], corresponding to the formation of a stable negative ion, this resonance is longest lived for a given value of the energy that corresponds to the EA of the system (for ground state collisions) or the BE of the metastable/excited anion. The important Regge Trajectories, viz. *Im* λ*_n_*(E) versus *Re* λ*_n_*(E), are also calculated in the CAM methods. They have been used to demonstrate that, at a low energy, relativistic and non-relativistic calculations yield essentially the same results [53].

### 3.2. Transition State Energy Barriers Calculation

The utility of the formed doubly-charged negative ions has been demonstrated in the catalysis of water oxidation into peroxide using the various doubly-charged systems indicated under the results. Figure 4, Figure 5 and Figure 6 demonstrate the Density Functional Theory (DFT)-calculated transition states. DFT and dispersion-corrected DFT approaches have been employed for the transition state evaluations. The geometry optimization of the structural molecular conformation utilized the gradient-corrected Perdew–Burke–Ernzerhof parameterizations [54] of exchange-correlation, as implemented in DMol3 [55]. A tolerance of 1 × 10^−3^ Ha was used, with a smearing value of 0.005 Ha. The DFT-calculated energy barriers reduction values in the oxidation of H_2_O to H_2_O_2_, catalyzed using the doubly-charged graphene, fullerene and atomic catalysts, are shown in Figure 4, Figure 5, Figure 6, Figure 7, Figure 8, Figure 9 and Figure 10.

## 4. Concluding Remarks

We have investigated, theoretically the catalytic effectiveness, during the oxidation of water into peroxide, of various doubly-charged negative ions of atomic metals and contrasted their performance with that of the doubly-charged fullerene and graphene molecular anions. Density Functional Theory TS calculations have found tunable energy activation barrier reduction values ranging from 0.030 eV to 2.070 eV, with Si^2−^, Pu^2−^, Pa^2−^ and Sn^2−^ being the best catalysts. This opens a new application opportunity for radioactive elements. The C_60_^2−^ reduces the standard C_60_^−^ transition state energy barrier significantly, while graphene increases it, behaving more like cationic systems that were previously discovered to increase the TS energy barriers in the synthesis of peroxide from H_2_O [56]. Our results should also be applicable in the petrochemical waste reduction of SO_2_ by CO catalyzed by the nanosystem Au_5_Cu^−^ [57], as well as in the conversion of methane to methanol without CO_2_ emission. Rank-ordered doubly-charged negative-ion catalysts are tabulated for easy selection for varied utilities according to their effectiveness, variation across charge states and systems. They reveal their tunable nature and wide applications, ranging from water purification to biocompatible antiviral and antibacterial sanitation systems. Indeed, the doubly-charged anionic catalysts that were obtained here offer a wide selection of atomic/molecular systems for the realization of inexpensive tunable dynamic water purification systems.

## Figures and Tables

**Table 1:** Rank-ordered activation energy barrier reduction, E(eV), for doubly-charged negative ionic systems, DCS(-2).

System	DCS(-2) E(eV)	System	DCS(-2) E(eV)	System	DCS(-2) E(eV)
Si	0.030	Pt	0.283	Ti	1.074
Pu	0.087	Pd	0.297	V	1.100
Pa	0.092	Ni	0.342	Gr	1.130
-	-	Rh	0.403	C_136_	1.440
At	0.098	Ag	0.429	C_20_	2.070
Sn	0.103	Os	0.443		
La	0.105	Co	0.541		
Zn	0.127	Au	0.575		
Eu	0.137	Ca	0.621		
Th	0.184	Cr	0.627		
U	0.244	Ce	0.710		
K	0.248	C_60_	0.784		
Tm	0.273	Fe	0.879		
Cu	0.277	Mn	1.033		

**Table 2:** Comparison of the activation energy barrier reduction, E(eV), between the doubly-charged systems, DCS(-2), and the singly-charged systems, SCS(-1).

System	DCS(-2) E(eV)	SCS(-1) E(eV)	System	DCS(-2) E(eV)	SCS(-1) E(eV)
Si	0.030	-	Rh	0.403	0.420
Pu	0.087	0.084	Ag	0.429	0.420
Pa	0.092	0.326	Au	0.575	0.400
At	0.098	0.172	Ca	0.621	1.188
Sn	0.103	1.210	Cr	0.627	-
La	0.105	0.035	C_60_	0.784	1.960
K	0.248	-	Gr	1.130	1.440
Pt	0.283	0.323	C_136_	1.440	0.680
Pd	0.297	0.200	C_20_	2.070	1.940
